# The influence of temperature environmental on performance of HNIW/FOX-7 based PBXs

**DOI:** 10.1038/s41598-022-08752-6

**Published:** 2022-03-23

**Authors:** Mengnan Zhou, Shusen Chen, Hui Chao, Na Wang, Bo Yan, Guanchao Lan, Shaohua Jin

**Affiliations:** 1 School of Materials Science and Engineering, Beijing Institute of Technology, Beijing, 100081 China; 2 Research Institute, Gansu Yin Guang Chemical Industry Group Co. Ltd, Baiyin, 730900 China

**Keywords:** Engineering, Materials science

## Abstract

During application, energetic materials may suffer different temperature environmental stimulation. In order to study the influence of temperature environmental on performance of HNIW/FOX-7 based PBXs, HNIW/FOX-7 based PBX modeling powders and PBX columns were treated by LT (low temperature), HT (high temperature), HLC (high-low temperature cycle) and HLS (high-low temperature shock). Then scanning electron microscope (SEM), infrared spectra (IR), X-ray diffraction (XRD) and differential scanning calorimetry (DSC) were used to study the variation of PBX modeling powders after LT, HT, HLC and HLS treatments; in addition, the mass, size and mechanical properties of PBX columns were characterized after different temperature adaptability treatments as well. The results indicate that the change ratios of mass and size of HNIW/FOX-7 based PBX columns are less than 1%, illustrating that mass and size of PBX columns are at acceptable level after different temperature adaptability treatments. The unevenness degree of the surface of PBX modeling powders followed the order of HLC > HT > LT > HLS, which agrees well with mass loss order. Moreover, IR and XRD results indicated that the molecular structure and crystal form of HNIW and FOX-7 did not change after different temperature adaptability treatments. Additionally, thermal stabilities of PBX modeling powders are decreased after different temperature adaptability treatments, among which HLS has the largest influence on HNIW/FOX-based PBX modeling powders. The compression strengths and elastic moduli of HNIW/FOX-based PBX columns are enhanced after different temperature adaptability treatments, among which the strength of PBX columns after HLC has the maximum increase, indicating that HLC has more significant effect on mechanical property.

## Introduction

2,4,6,8,10,12-Hexanitro-2,4,6,8,10,12-hexaazaisowurtzitane (HNIW) with cage structure has been widely used as the main explosive in many high energy polymer bonded explosives (PBXs) formulations^1–3^ because of its high energy density, high detonation pressure and high detonation velocity^4,5^. However, energetic materials with high energy often accompany with high sensitivity and low security. High energy of HNIW based PBXs bring high sensitivity and hazards as well. Nowadays, the sensitivity and vulnerability of high energy explosives are expected to be reduced for enhancing the security of modern weaponry. In order to achieve this goal, using insensitive explosive such as 1,1-diamino-2,2-dinitroethylene (FOX-7) to replace some HNIW to prepare HNIW/FOX-7 based PBXs is a good technique. By adjusting the content of FOX-7, the energy and security of HNIW/FOX-7 based PBXs can be balanced^6,7^.

Explosives will undergo various temperature environments during their storage, transportation and application. The complex temperature environments may change the mass, size and detonation properties of munitions, which may produce damage and aging inside munitions and exert effects on energy, security and mechanical properties of PBXs, and then produce negative influence on overall functionality of weapon system^8^. In addition, HNIW have more than one kind of crystalline form, and different crystalline form can be converted to each other at high temperature, and FOX-7 has the same phenomenon. The various temperature environments may change the crystalline form of HNIW and FOX-7, which may result in great variation of properties of HNIW/FOX-7 based PBXs. Therefore, the investigation of temperature adaptability of HNIW/FOX-7 based PBXs is imperative.

In order to study the influence of temperature environmental on performance of HNIW/FOX-7 based PBXs. LT (low temperature), HT (high temperature), HLC (high-low temperature cycle) and HLS (high-low temperature shock) are first used to treat HNIW/FOX-7 based PBX modeling powders and PBX columns. After LT, HT, HLC and HLS treatments, scanning electron microscope (SEM), infrared spectra (IR), X-ray diffraction (XRD) and differential scanning calorimetry (DSC) were used to study the variation of PBX modeling powders. In addition, the mass, size and mechanical properties of PBX columns were characterized after HT, LT, HLC and HLS treatments as well. By comparing the variation SEM, IR, XRD, DSC, mass, size and mechanical properties after different temperature adaptability treatments, the temperature adaptability of HNIW/FOX-7 based PBXs are obtained.

## Experimental details

### Preparation of HNIW/FOX-7 based PBX samples

**Figure 1:**
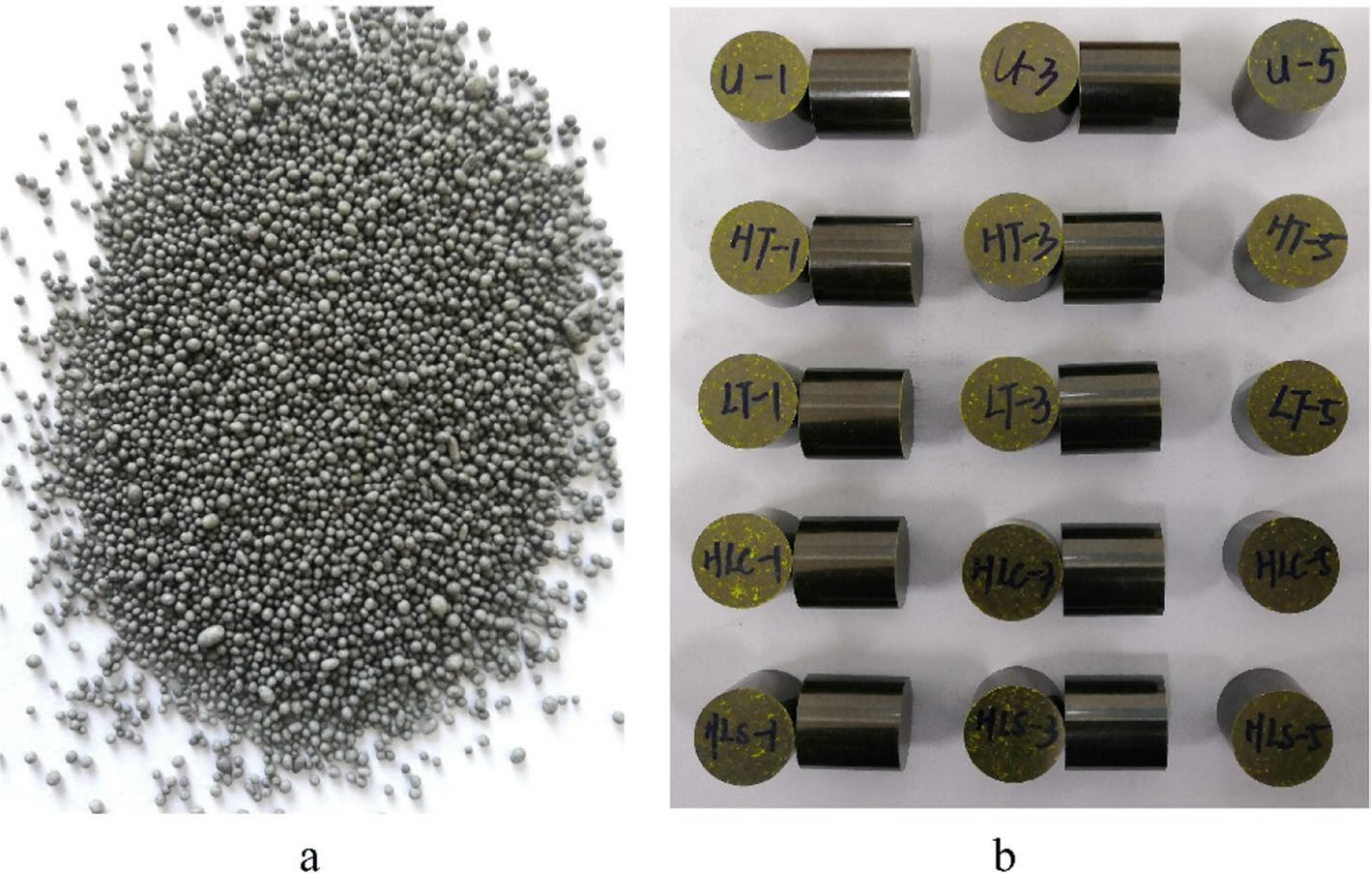
(**a**) HNIW/FOX-7 based PBX modeling powders, (**b**) HNIW/FOX-7 based PBX columns.

Water suspension method^9,10^ was adopted to prepare HNIW/FOX-7 based PBX modeling powders (47 wt% HNIW, 47 wt% FOX-7, 5 wt% binder system, 0.5 wt% wax, 0.5 wt% graphite). The PBX modeling powders prepared in this study are displayed in Fig. 1a. Then, the modeling powders were further pressed to *Φ* 20 × 20 mm PBX columns using hydraulic press with the temperature and loading rate of 25 °C and 1 kN s^−1^ respectively. When the pressure of hydraulic press reached the maximum values (2000 kg cm^−2^), maintain the maximum pressure for 10 min. Adopting these conditions, the real density of HNIW/FOX-7 based PBX columns could reach 97% of the theoretical maximum density. The PBX columns prepared in this study are displayed in Fig. 1b.

### Temperature adaptability treatments

**Figure 2:**
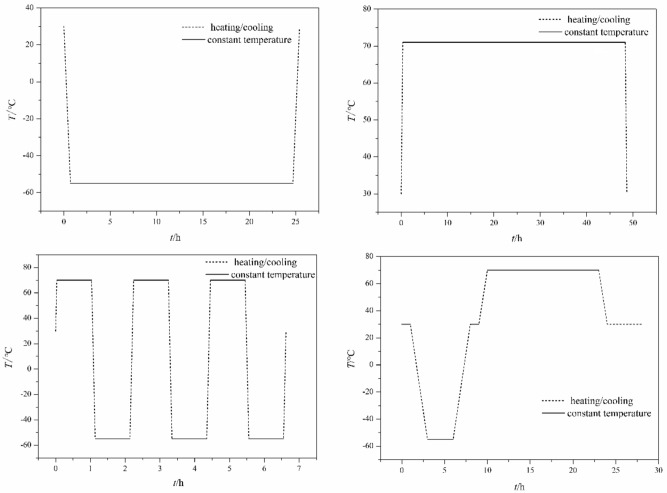
Temperature variation settings of different temperature adaptability treatments.

In this study, using constant humidity-temperature chamber (QS-GS100FB), HNIW/FOX-7 based PBXs are treated under LT, HT, HLS and HLC, respectively^11,12^. Temperature variation settings of LT, HT, HLS and HLC are shown in Fig. 2.

At ambient temperature, 10 g PBX modeling powders and 5 PBX columns were placed into constant humidity-temperature chamber. The temperature of chamber was froze to − 55 °C with a cooling rate of 2 °C min^−1^ and maintained for 24 h to study the effect of LT on the performance of HNIW/FOX-7 based PBX. The temperature of chamber was heated to 71 °C with a heating rate of 2 °C min^−1^ and maintained for 48 h to study the effect of HT on the performance of HNIW/FOX-7 based PBX. The temperature of chamber was heated to 71 °C and maintained for 1 h, and then the temperature was froze to − 55 °C and maintained for 1 h. During the test, the heating and cooling rate was controlled at 20 °C min^−1^. Repeat this cycle three time to study the effect of HLS on the performance of HNIW/FOX-7 based PBX. HNIW/FOX-7 based PBX samples were placed in temperature cycle between − 55 and 71 °C for five cycles to study the effect of HLC on PBX performance. For each cycle, the room temperature (30 °C), low temperature (− 55 °C) and high temperature (71 °C) were maintained for 2, 3 13 h respectively. The heating and cooling rate was controlled at 0.67 °C min^−1^ during temperature variation process.

### Characterizations

#### Mass and size of PBX columns

After LT, HT, HLS and HLC treatments, the mass and size (diameter and height) of the PBX columns were measured using analytical balance and screw micrometer, respectively. The average variation ratio of mass and size of 5 PBX columns were obtained.

#### Surface morphology

After LT, HT, HLS and HLC treatments, the surface morphology of PBX modeling powders was obtained by scanning electron microscope (SEM, MIRA3 XM, Tescan Co., Ltd., Brno, Czech Republic).

#### Infrared spectrum (IR)

After LT, HT, HLS and HLC treatments, IR spectra of PBX modeling powders were recorded using KBr plates on a Nicolet 6700 spectrometer produced by Thermo Fisher Scientific. The wavenumber ranges from 4000 to 400 cm^−1^, and the tests were carried out at room temperature.

#### X-ray diffraction (XRD)

After LT, HT, HLS and HLC treatments, X-ray powder diffraction of PBX modeling powders were collected on a Rigaku Ultima IV which was made by Beijing Glory Leader Technology Co., Ltd. The test voltage and electric current were 40 kV and 44 mA, respectively. Scanning angle ranges from 10° to 70° with the scanning speed of 2°/min.

#### Thermal decomposition

After LT, HT, HLS and HLC treatments, thermal decomposition behavior of PBX modeling powders was measured using differential scanning calorimeter (DSC 200 F3, aluminum crucible with a pin hole cover). The sample with the mass of 0.70 ± 0.01 mg was heated from 50 to 320 °C under nitrogen with a flow rate of 70 mL min^−1^ with a heating rate of 0.5, 1, 1.5 and 2 °C min^−1^, respectively. Each sample was analyzed three times.

Flynn–Wall–Ozawa (FWO)^13^ methods, expressed in Eq. (1), was adopted to calculate the kinetics parameters of the main exothermic decomposition reactions.

1 $$\mathrm{ln}\beta =\mathrm{lg}\left[\frac{{k}_{0}{E}_{a}}{Rg\left(\alpha \right)}\right]-5.331-1.052\frac{{E}_{a}}{R{T}_{\mathrm{P}}}$$

where *β* is the heating rate (°C min^−1^), *T*_p_ is the peak temperature (K), *E*_a_ is the apparent activation energy (kJ mol^−1^), *A* is the pre-exponential factor (s^−1^), *R* is the gas constant valued as 8.314 J mol^−1^ K^−1^, *α* is the conversion degree that was the mass ratio of the reacted substance to the raw, and *G*(*α*) is the integral mechanism function.

#### Adiabatic accelerating thermal decomposition

After LT, HT, HLS and HLC treatments, adiabatic thermal decomposition behavior of PBX modeling powders was studied using accelerating rate calorimeter (ARC, NETZSCH 254) instrument. 110 ± 1 mg samples, spherical hastelloy C vessel, and ‘heat-wait-search’ procedure were used during the measurement. The temperature range of ARC experiment is between 50 and 230 °C. Based on the measured results, the kinetics parameters and mechanism function of PBX modeling powders adiabatic thermal decomposition are obtained.

#### Mechanical property

After LT, HT, HLS and HLC treatments, according to GJB 772A-97^14^, the compression strength (*σ*_c_) and elastic modulus (*E*) of PBX columns are analyzed using electromechanical universal testing machine (CMT4502). Each PBX column is imposed a quasi-static compression load along the axial with a loading rate of 0.5 mm min^−1^ until electromechanical universal testing machine reach the maximum load (4.5 kN) or PBX column was damaged. Then *E* and *σ*_c_ could be calculated by the following two equations,

2 $$\varepsilon =\frac{\sigma }{E}+A{\sigma }^{m}$$

3 $${\sigma }_{\mathrm{c}}=\frac{4P}{{\pi d}^{2}}$$

where *ε* and *σ* are strain and stress respectively, *A* and *m* are model parameters, *P* is the compression load and *d* is the diameter of PBX column.

## Results and discussion

### Mass and size

Table 1 lists the change ratios of mass and size (height and diameter) of HNIW/FOX-7 based PBX columns. The results illustrate that the change ratios of mass and size (height and diameter) of PBX columns are within 1%. Based on American military standard MIL-STD-1751^15^, the mass and size of PBX columns are still at acceptable level after different temperature adaptability treatments.

**Table 1:** Variation ratios of mass and size of the HNIW/FOX-7 based PBX columns.

Test	(Δ*d*/*d*)/%	(Δ*h*/*h*)/%	(Δ*ρ*/*ρ*)/%	(Δ*m*/*m*)/%
HT	− 0.099	0.078	0.213	− 0.093
LT	− 0.050	0.000	0.152	− 0.052
HLC	− 0.129	0.185	0.182	− 0.108
HLS	− 0.139	− 0.039	0.356	− 0.040

After LT, HT, HLS and HLC treatments, the mass of each PBX column is declined, and the variation ratio of the mass of PBX columns after different temperature adaptability treatments follow the order of HLC > HT > LT > HLS, which is associated with the treatment time of temperature adaptability treatments. The variation of the size of PBX columns is more complex than that of mass. It can be concluded that all the diameters are decreased, but the heights are not totally increased or decreased. On the one hand, the mass loss of PBX columns may cause the variation of size. On the other hand, the variation of temperature affects the motion ability of PBX molecules may cause the variation of size as well. The densities of PBX columns are increased after different temperature adaptability treatments, in that the internal stress of PBX columns are released resulting in the decrease of volume. The change ratios of PBX columns after different temperature adaptability treatments are far less than the failure criterion. Therefore, the influence of LT, HT, HLS and HLC on the mass and size of HNIW/FOX-7 based PBX columns can be neglected.

### Surface morphology

**Figure 3:**
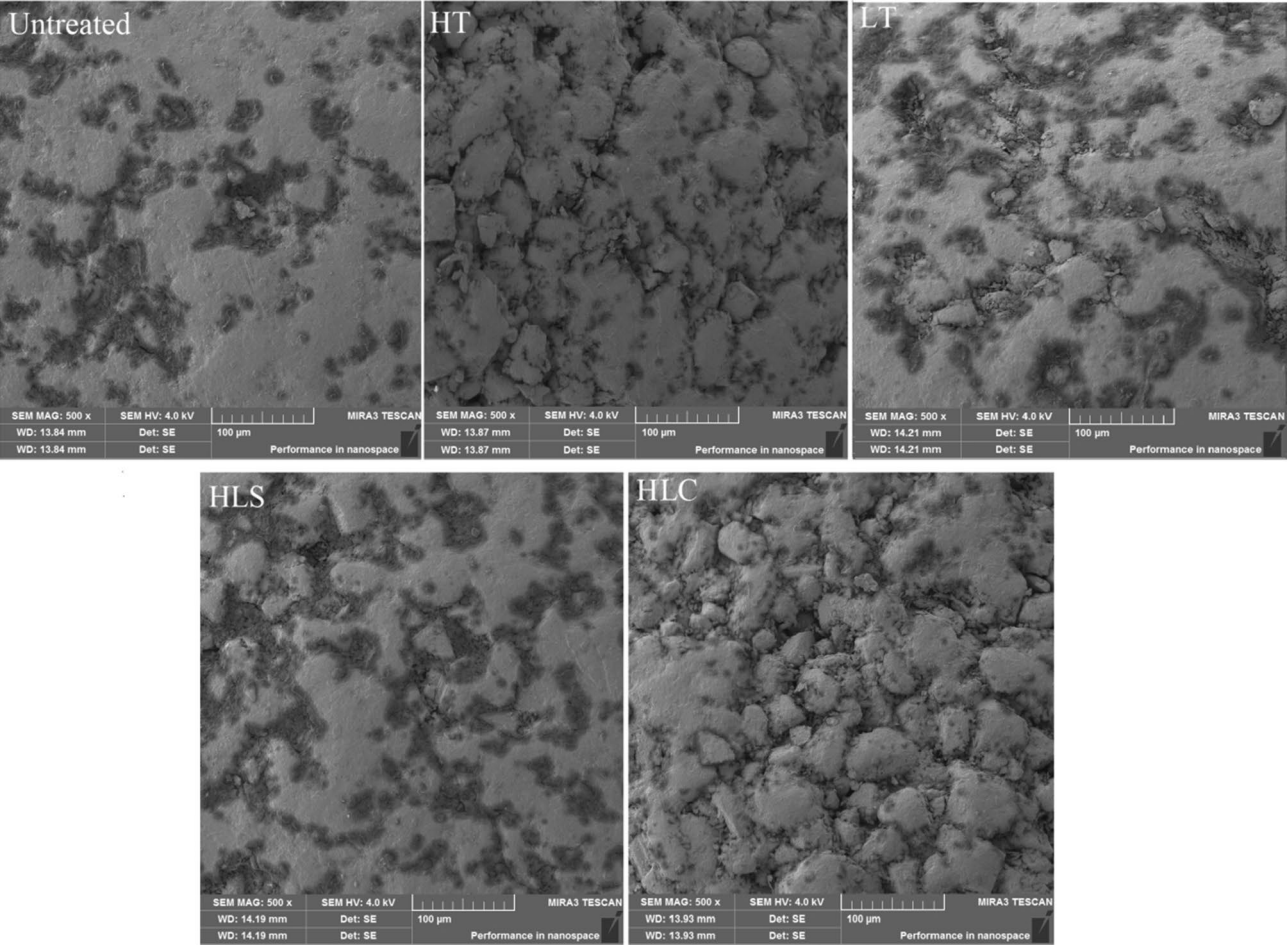
Surface morphology of the HNIW/FOX-based PBX modeling powders.

SEM is used to study the surface morphology of HNIW/FOX-based PBX modeling powders. Figure 3 displays the surface morphology of PBX modeling powders before and after LT, HT, HLS and HLC treatments. After different temperature adaptability treatments, the surface of PBX modeling powders become uneven and emerge many cavities, which is probably caused by the sublimation and gasification of the additives. In addition, it is can be concluded from Fig. 3 that the unevenness degree of the surface of PBX modeling powders follows the order of HLC > HT > LT > HLS, which agrees well with variation ratios of mass loss.

### Molecular structure

**Figure 4:**
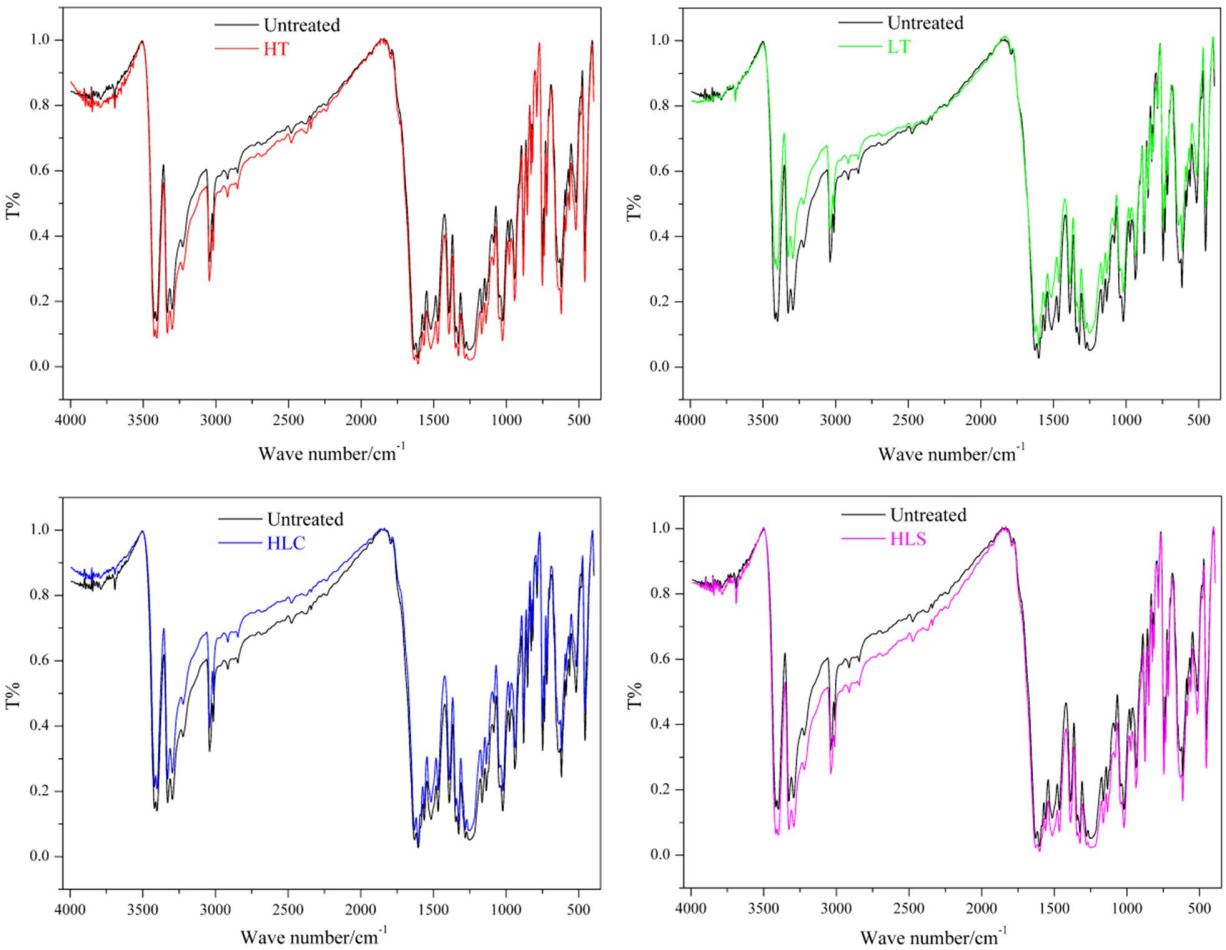
IR spectroscopies of HNIW/FOX-based PBX samples.

To investigate whether the structures of HNIW/FOX-based PBX samples have changed after LT, HT, HLS and HLC treatments, the samples are characterized by IR spectroscopy (Fig. 4). It can be concluded from Fig. 4 that after different temperature adaptability treatments all IR spectroscopies are consistent with the untreated sample, indicating that the structures of HNIW/FOX-based PBX samples have not changed.

### Crystalline form

**Figure 5:**
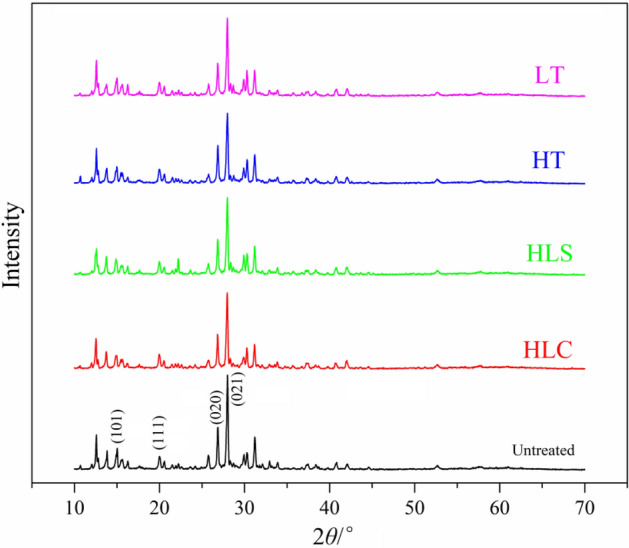
XRD spectroscopies of HNIW/FOX-based PBX samples.

HNIW and FOX-7 exist polymorphs^16–18^, and their crystalline form may transform at high temperature. To research whether the crystalline form of HNIW and FOX-7 in HNIW/FOX-7 based PBX has changed after LT, HT, HLS and HLC treatments, the samples are characterized by XRD (Fig. 5). Figure 5 shows that after different temperature adaptability treatments all XRD spectroscopies are consistent with the untreated sample, indicating that the crystal form of HNIW and FOX-7 in HNIW/FOX-7 based PBX samples has not changed.

### Mechanical properties

**Figure 6:**
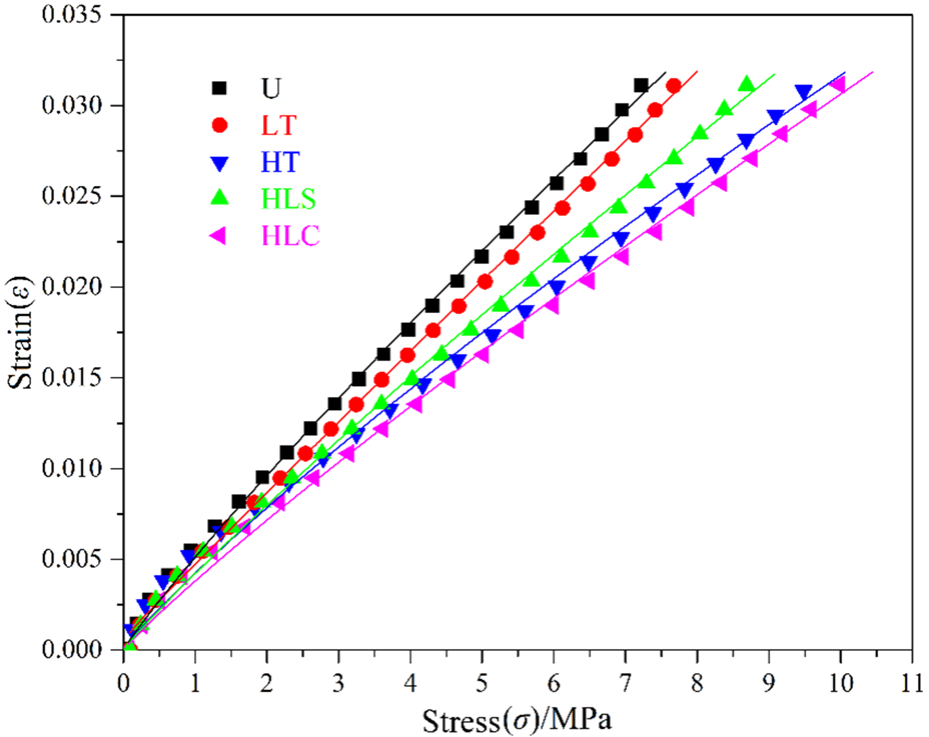
Stress–strain curves of HNIW/FOX-based PBX columns.

After different temperature adaptability treatments, the compression strengths and elastic moduli of PBX columns are investigated. The stress–strain curves of PBX columns are displayed in Fig. 6, and the calculated compression strengths and elastic moduli are listed in Table 2.

**Table 2:** Compression strengths and elastic moduli of the HNIW/FOX-7 based PBX columns.

Treatments	*σ*_c_/MPa	*E*/MPa
Untreated	8.07	2.30
HT	10.96	3.01
LT	8.38	2.47
HLC	11.14	3.17
HLS	10.11	2.78

It can be concluded from Table 2 that the compression strengths and elastic moduli of the PBX columns are all increased after HT, LT, HLC and HLS, indicating the enhance of rigidity of PBX columns. The variation of *σ*_c_ and *E* after LT is relatively small, demonstrating that the effects of low temperature on HNIW/FOX-7 based PBX columns are small. However, the influence of HLC on HNIW/FOX-7 based PBX columns are significant. Although the content of binder system is low in this HNIW/FOX-7 based PBX formulation, it may repair some defects induced by pressing because of its fluidity and spreadability during storage, thus increasing the mechanical properties of the PBX columns. In addition, oxidative crosslinking reaction of binder may occur after temperature adaptability tests under the influence of temperature, which increases the crosslinking point of the polymer network formed after reaction and then raise the crosslinking density, and finally improves compression properties and elastic moduli.

### Thermal decomposition

**Figure 7:**
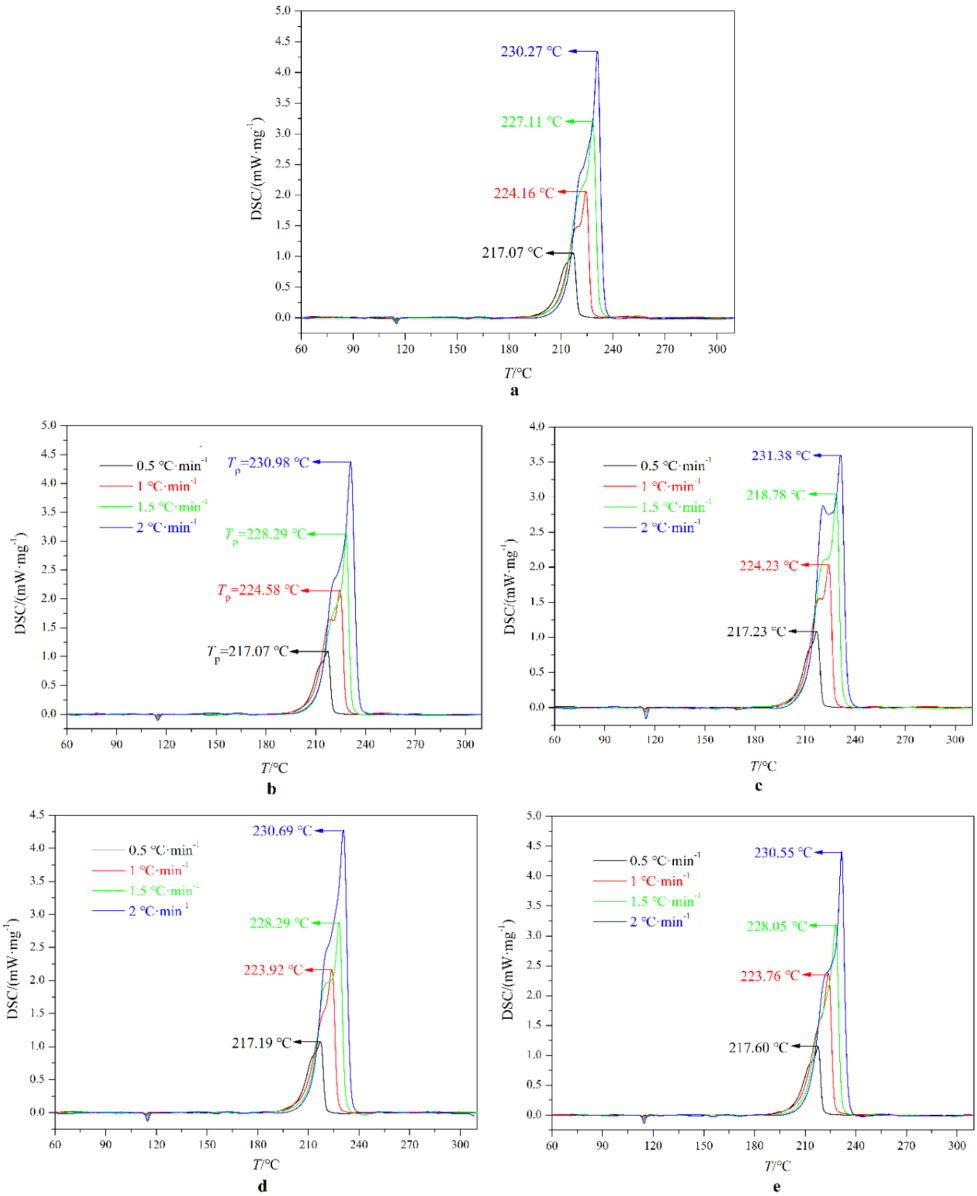
DSC curves of HNIW/FOX-7 based PBX samples. (**a**) Untreated, (**b**) HLC treatment, (**c**) LT treatment, (**d**) HT treatment, (**e**) HLS treatment.

After different temperature adaptability treatments, thermal decomposition performance of the HNIW/FOX-7 based PBX samples are studied by DSC. The measured DSC curves are displayed in Fig. 7. The initial decomposition temperature (*T*_o_), peak temperatures (*T*_p_) and released heat (Δ*H*) obtained by DSC measurements are summarized in Table 3.

**Table 3:** The initial decomposition temperature (*T*_o_), peak temperatures (*T*_p_) and released heat (Δ*H*) of PBX samples.

Treatments	*β*/°C·min^−1^	*T*_o_/°C	*T*_p_/°C	Δ*H*/J·g^−1^
Untreated	0.5	204.54	217.07	− 1608
	1.0	209.11	224.16	− 1647
	1.5	212.09	227.11	− 1696
	2.0	213.93	230.27	− 1778
HLC	0.5	204.33	217.07	− 1590
	1.0	207.82	224.58	− 1612
	1.5	210.06	228.29	− 1677
	2.0	212.19	230.98	− 1744
HLS	0.5	203.79	217.60	− 1583
	1.0	206.89	223.76	− 1626
	1.5	210.62	228.05	− 1690
	2.0	212.22	231.55	− 1748
LT	0.5	203.59	217.23	− 1587
	1.0	206.97	224.23	− 1634
	1.5	209.65	228.78	− 1686
	2.0	212.55	231.38	− 1756
HT	0.5	203.81	217.19	− 1551
	1.0	207.04	223.92	− 1624
	1.5	210.59	228.29	− 1676
	2.0	212.22	230.69	− 1759

Table 3 shows that at the same heating rate *T*_o_ and Δ*H* of PBX samples are decreased after HT, LT, HLC and HLS treatments, illustrating that the thermal insulation effect of binder system and desensitizer on PBX samples may be weakened, resulting in a decrease of PBX samples thermal stability. The decreasing Δ*H* reveals that the detonation heat may decline after temperature adaptability treatment. The variation of *T*_p_ of PBX samples is more complicated, but the variation ratios are lower than 2 °C illustrating that the thermal stabilities of HNIW/FOX-7 based PBXs unchanged after diifernt temperature adaptability treatments.

The kinetics parameter (*E*_a_) of HNIW/FOX-7 based PBX samples are calculated using FWO. The calculated *E*_a_ and correlation coefficient (*R*^2^) are listed in Table 4. We can find that the *R*^2^ are higher than 0.99, illustrating the calculated kinetics parameters are accurate. Generally, materials with higher value of *E*_a_ will be more stable. Table 4 shows that the *E*_a_ declined after different temperature adaptability treatments, which indicates a probable decline of the stability of HNIW/FOX-7 based PBXs.

**Table 4:** The kinetic parameters of HNIW/FOX-7 based PBX samples.

Sample	*E*_a_/kJ mol^−1^	*R*^2^
Untreated	41.906	0.9975
HLC	39.269	0.9982
HLS	39.740	0.9987
HT	38.431	0.9989
LT	40.072	0.9985

### Adiabatic accelerating thermal decomposition

**Figure 8:**
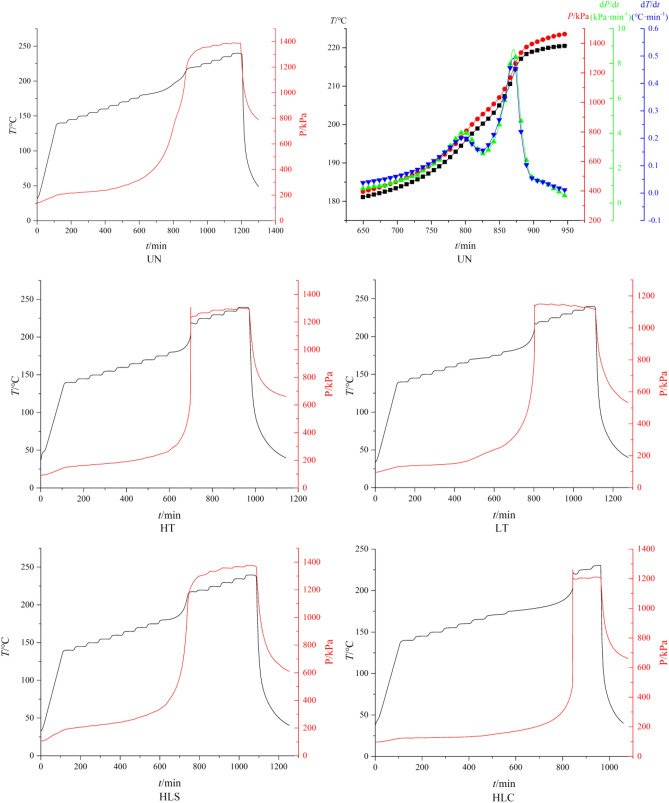
ARC measured results, and the variation of *P*, *T*, d*P* and d*T* versus *t* during the adiabatic decomposition process of untreated samples.

The adiabatic decomposition properties of HNIW/FOX-7 based PBX samples are studied by ARC instrument after different temperature adaptability treatments. The measured initial decomposition temperature (*T*_o_), final decomposition temperature (*T*_f_), adiabatic temperature rise (Δ*T*_ad_), maximum pressure (*P*_m_) and released heat (*Q*) are listed in Table 5. The measured ARC curves are displayed in Fig. 8, and the variation of temperature (*T*), pressure (*P*), temperature change rate (d*T*/d*t*) and pressure change rate (d*P*/d*t*) with time (*t*) during the adiabatic decomposition process of untreated samples are depicted in Fig. 8 as well.

**Table 5:** ARC measured adiabatic thermal decomposition parameters.

Parameters	Untreated	LT	HT	HLC	HLS
*T*_0_/°C	181.66	181.28	181.32	181.59	180.54
*T*_f_/°C	222.14	218.81	219.94	220.46	219.43
Δ*T*_ad_/°C	40.48	37.53	38.62	38.87	38.89
*P*_m_/kPa	1422.36	1354.27	1310.10	1248.18	1394.01
*Q*/J g^−1^	37.70	34.95	35.97	36.20	36.22

ARC measured results illustrate that the initial decomposition temperature of each sample is around 180 °C demonstrating that temperature adaptability treatments have few influences on the adiabatic decomposition stability of HNIW/FOX-7 based PBXs. In addition, the Δ*T*_ad_, *P*_m_ and *Q* are decrease after HLC, HLS, HT and LT treatments, but the decrease ratios are low, illustrating that temperature adaptability treatments have few influences on the adiabatic decomposition stability of PBXs as well.

According to ARC measured results, the activation energies (*E*_a_), pre-exponential factors (*A*) and mechanism functions (*f*(*α*)) of HNIW/FOX-7 based PBXs adiabatic decomposition are calculated using mechanism functions method^19–22^. The calculated results are summarized in Table 6.

**Table 6:** The calculated *E*_a_, *A* and *f*(*α*) of adiabatic decomposition.

Samples	*E*_a_/kJ mol^−1^	*A*/s^−1^	*f*(*α*)	*R*^2^
Untreated	290.458	3.594 × 10^30^	(1 − *α*)^2^	0.992
HLC	273.162	8.767 × 10^26^	(1 − *α*)^2^	0.991
HLS	281.679	1.063 × 10^27^	(1 − *α*)^2^	0.984
HT	278.851	8.902 × 10^26^	(1 − *α*)^2^	0.986
LT	284.713	9.134 × 10^27^	(1 − *α*)^2^	0.993

It can be deduced from Table 6 that *f*(*α*) is still (1-*α*)^2^ after different temperature adaptability treatments, illustrating HLC, HLS, HT and LT treatments wouldn’t change the mechanism functions of HNIW/FOX-7 based PBXs. *E*_a_ decreased 17.30 kJ mol^−1^ (5.95%), 8.78 kJ mol^−1^ (3.02%), 11.61 kJ mol^−1^ (4.00%) and 5.75 kJ mol^−1^ (1.98%), respectively after HLC, HLS, HT and LT treatments. It can be also concluded that the variation ratios of *E*_a_ is related to the treatments time of high temperature. After HLC, HLS, HT and LT treatments, the variation ratios of *E*_a_ are lower than 20%, which are still in an acceptable level according to GJB 772A-97.

## Conclusions

In this study, HNIW/FOX-7 based PBX samples were treated by LT, HT, HLC and HLS. The mass, size, surface morphology, molecular structure, crystalline form, mechanical property and thermal decomposition are characterized to study temperature environmental adaptability of HNIW/FOX-7 based PBXs. The change ratios of mass and size (diameter and height) of HNIW/FOX-7 based PBX columns are lower than 1%, indicating that PBX columns are still at an acceptable level after different temperature adaptability treatments. The surface morphology of PBX samples become uneven and emerge many cavities, which is probably caused by the sublimation and gasification of the additives. The characterization results of IR and XRD demonstrate that the structure and crystal form of HNIW/FOX-7 based PBXs have not changed after temperature adaptability treatments. The compression strengths and elastic moduli of PBX columns are increased after HT, LT, HLC and HLS treatments, indicating the enhance of rigidity of PBX columns. HT, LT, HLC and HLS treatments have few influences on the initial decomposition temperatures, activation energies and mechanism functions of HNIW/FOX-7 based PBXs, indicating that the thermal stabilities of PBXs are almost unchanged.

Above all, after HT, LT, HLC and HLS treatments the performances of HNIW/FOX-7 based PBXs have no significant change, illustrating the temperature environmental adaptabilities of HNIW/FOX-7 based PBXs are adaptable.
